# Intratesticular Versus Intrafunicular Lidocaine to Reduce Perioperative Nociception and Immunological Response in Ponies Undergoing Field Castration

**DOI:** 10.3390/vetsci9120664

**Published:** 2022-11-28

**Authors:** Cecilia Vullo, Rosalia Crupi, Rosanna Di Paola, Salvatore Cuzzocrea, Enrico Gugliandolo, Vito Biondi, Giuseppe Catone

**Affiliations:** 1 Department of Chemical, Biological, Pharmaceutical and Environmental Sciences, University of Messina, Viale Ferdinando Stagno D’Alcontres, 31, 98166 Messina, Italy; 2 Department of Veterinary Sciences, University of Messina, Polo SS. Annunziata, 98169 Messina, Italy

**Keywords:** castration, anaesthesia, lidocaine, cytokines

## Abstract

**Simple Summary:**

Castration is a procedure performed routinely in male ponies to facilitate handling and husbandry and reduce aggression and other undesirable behaviours associated with testosterone. It can be performed in field conditions under general anaesthesia. The procedure is associated with a significant degree of pain that requires adequate analgesics, such as local anaesthetics, as an adjunct to general anaesthesia. The current study aimed to clarify the effects of two different local anaesthesia techniques with lidocaine 2% on the plasmatic concentrations of TNF-α and IL-6 in ponies submitted to field castration. The hypothesis was that the two different locoregional anaesthetic protocols could impact the immunological response and the postoperative outcome.

**Abstract:**

The aim of this study was to evaluate the impact of intratesticular or intrafunicular lidocaine to reduce perioperative nociception and cytokine release in ponies undergoing field castration under total intravenous anaesthesia. Before castration, one group was injected with intrafunicular (FL) lidocaine and the other received intratesticular (TL) lidocaine. All ponies were premedicated with acepromazine (0.05 mg/kg) intramuscularly. Twenty minutes after the administration of acepromazine, xylazine (1 mg/kg) and butorphanol (0.02 mg/kg) were administered intravenously. Lidocaine 2% was given 1 mL/100 kg intrafunicularly in the FL groups or 2 mL/100 kg intratesticularly on each testicular side for TL. Surgery was performed by the same team of two experienced surgeons using Serra’s emasculator and an open technique was used for all ponies in order to promote postoperative drainage. In this study, we focused on the plasmatic levels of TNF-α and IL-6. The results from this study showed a significant difference in plasmatic concentrations of TNF-α and IL-6 between the two different locoregional anaesthetic protocols. Taken together, the results suggest that the intrafunicular lidocaine locoregional anaesthesia could be a useful technique in the anaesthesia protocol for field pony castration.

## 1. Introduction

During and after surgery, the local and systemic inflammatory response plays a key role in the positive outcome of surgery. Although a “proper” inflammatory response is a physiological response to the surgical tissue injury, an unbalanced inflammatory response affects wound healing and is also involved in the development of postoperative complication [1]. The cytokine network is closely related to tissue homeostasis, inflammatory response, immune response, and neuroendocrine system activation. To date, it is widely recognised that high levels of inflammatory cytokines may also be associated with postoperative pain sensitisation, fatigue, and systemic inflammation [2]. Castration is one of the most common surgical procedures performed in equine practice and it is performed to reduce or prevent aggressive behaviour, to treat testicular trauma or neoplasia, or inguinal herniation [3]. Currently, greater attention is paid to the control of pain and the improvement of animal well-being, and in the pharmacological protocol for pain control during surgical procedures [4]. The procedure may be performed in a standing, sedated animal or in a recumbent animal under general anaesthesia [5,6,7,8]. Ideally, a balanced anaesthetic protocol helps to prevent nociception during surgery and postsurgery and prevents immune system activation, inflammation, and pain sensitisation [9]. Several studies suggest that locoregional anaesthetic techniques provide a useful contribution in anaesthetic protocols for surgical procedures. As already reported, the use of local lidocaine is able to reduce the dose of intravenous anaesthetic [10,11]. However, to date there are few data on the use of the different locoregional anaesthetic techniques in pony castration. Thus, the objective of this study was to evaluate the effects of intratesticular or intrafunicular lidocaine to reduce perioperative nociception and cytokine release in ponies undergoing field castration under total intravenous anaesthesia.

The hypothesis of the study was that with intrafunicular local anaesthesia for field castration, less reaction to surgical stimulation would be detectable and that postoperative pain and cytokine production would be reduced compared to intratesticular local anaesthesia. The assessment of equine pain after surgery is a difficult challenge and there is no gold standard technique to evaluate it [12]. In this study, we focused on the changes of the plasmatic levels of Interleukin-6 (IL-6) and Tumor Necrosis Factor alpha (TNF-α) pre- and postsurgery. We focused on IL-6 and TNF-α as the two major proinflammatory cytokines that play a key role in several aspects of the immune inflammatory response [13,14]. Evidence suggested that levels of proinflammatory cytokines such as TNF-α and IL-6 are related to increases in nociception and pain sensitisation [15]. IL-6 is widely recognised as an important mediator in the wound healing process, and local and systemic inflammatory response. IL-6 exhibits a pleiotropic effect which involves an important activation of the immune system response [16], studies demonstrate that elevated IL-6 levels in the postoperative periods are associated with a negative outcome of surgery and with an increased systemic inflammatory response [17]. TNF-α, considered a major proinflammatory mediator, exerts a physiological role in host defence, tissue repair, and wound healing processes. High levels of TNF-α are related to a negative surgery outcome in terms of tissue and systemic inflammation and nociception sensitisation [18,19]. The modulation of the levels of these two proinflammatory cytokines could be a key step in reduction of both local and systemic nociception as it is widely recognised that the interaction between nociceptor neurons and immune system is a key event in both acute and chronic inflammatory response [20].

## 2. Materials and Methods

### 2.1. Animals and Study Design

**Figure 1:**
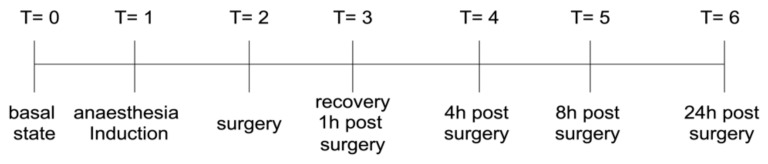
Experimental design.

This study was approved by the Bioethics Committee of the Department of Veterinary Sciences of the University of Messina according to the Good Scientific Practice Guidelines and the European legislation, EU Directive 2010/63 (Ethical approval code N. 078/2022). Fourteen healthy adult ponies, American Society of Anesthesiologists (ASA) physical status 1, aged from 3–6 years, with body weight ranging from 180–240 kg, were involved in the study. Exclusion criteria included ASA physical status ≥2, intractable behaviour, neurological or neuromuscular disease, and the presence of undescended testis. Ponies had free access to water but fasted 6 h prior to surgery. Before surgery, intramuscular (IM) benzylpenicillin procaine and dihydrostreptomycin (ì9.000 U/kg and 11.25 mg/kg; Combiotic, Huvepharma; Garessio, Italy) and IV flunixin meglumine (1.1 mg/kg; Flunixin, Vetoquinol; Bertinoro, Italy) were administered. Body weight was estimated on the bases of the body length and chest girth [21]. Before administering any drug, rectal temperature (RT baseline, °C), heart rate (HR, beats/min), and respiratory rate (RR, breaths/min) were recorded. RT was measured using a digital thermometer, HR was measured by auscultation, using a stethoscope, and RR by observing thoracic excursion. The animals were randomly assigned to one of two treatment groups: (A) group FL: intrafunicular lidocaine; (B) group TL: intratesticular lidocaine. A manual randomisation technique was used to assign animals into the groups using an envelope method. Each treatment group was composed of eight ponies. Figure 1 shows the experimental timeline of the blood sample withdrawal.

### 2.2. Anaesthesia Premedication, Induction, and Surgery

Following aseptic techniques, a 14-gauge, 13 cm catheter was placed in the external jugular vein, through a lidocaine injected subcutaneously 5 min before. All ponies were premedicated with acepromazine (0.05 mg/kg; Prequillan, Fatro; Ozzano, Italy) intramuscularly. Twenty minutes after the administration of acepromazine, xylazine (1 mg/kg; Rompun, Bayer; Milano, Italy) and butorphanol (0.02 mg/kg; Dolorex, Animal Health; Segrate, Italy) were administered intravenously. Sedation was considered adequate based on a 4-point sedation score (score 0, poor: fully responsive to environment, lips apposed, no lowering of head, no drooping of the ears; score 1, mild: still responsive to environment, slight separation of the lower lip, slight lowering of the head, slight drooping of the ears; score 2, good: no response to environment, separation of the lower lip, lowering of the head, drooping of the ears; score 3, heavy: no response to environment, extreme lip separation, pronounced loss of postural tone and ataxia, pronounced separation of the ear tips) [22]. An additional dose of xylazine (0.3 mg/kg IV) was administered if the sedation score was 0 or 1. Induction of anaesthesia was performed by diazepam (0.05 mg/kg; Ziapam, Dechra; Torino, Italy) and ketamine (2.2 mg/kg; Nimatek, Dechra; Torino, Italy) intravenously. Induction was evaluated based on a 4-point induction score (score 0, poor: ataxia, excitement without falling down; score 1, sufficient: ataxia, fall with paddling and attempts to stand up; score 2, good: no ataxia, able to move 1 or 2 steps with no paddling after falling down; score 3, very good: no ataxia, smoothly falling down to the ground) [22]. If the induction score was 0 or 1 an additional dose of ketamine (0.1 mg/kg IV) was administered. Once the ponies were recumbent, they were positioned in right lateral recumbency with the left hind limb elevated, and the head and neck extended to maintain a patent airway. Immediately, an infusion of guaifenesin (Knock-out, ACME; Castelnuovo del Garda, Italy), ketamine, and xylazine was started to maintain anaesthesia. The final solution of 50 mg/mL guaifenesin, 2 mg/mL ketamine, and 1 mg/mL xylazine was created by adding 1 g of ketamine and 500 mg of xylazine to a 500 mL bag of 5% guaifenesin. The infusion was started at one drop/second, and it was decreased or increased based on monitoring of eye signs, muscle relaxation of the neck, RR and pattern, and reaction to surgical stimulation. Lidocaine 2% (Lidocaina 2%, Esteve; Milano, Italy) was given 1 mL/100 kg intrafunicularly in the FL groups or 2 mL/100 kg intratesticularly on each testicular side for TL. Surgery was performed by the same team of two experienced surgeons using Serra’s emasculator, and an open technique was used for all ponies to promote postoperative drainage. Arterial oxygen saturation (SpO_2_; Nellcor Portable SpO_2_, Covidien), HR, and RR were recorded during surgical preparation and every 5 min during the surgery (Table 1). The anaesthesia time (time from induction to the end of the infusion), time to carry out the surgery (aseptic preparation, spermatic cord infiltration, and surgery), and time from the end of the infusion to standing (recovery time) were recorded. Recovery was assisted with a rope applied only to the head. If additional analgesia was required, it was provided with dipyrone followed by flunixin meglumine (within 15 min if pain persisted).

### 2.3. Cytokine Plasma Concentrations

For determination of cytokines, at each time point (Figure 1) venous blood samples were collected by venous catheter. The blood was collected in heparinised tubes (Vacutainer). Once harvested, blood samples were centrifuged (400× *g* for 10 min at 4 °C) within 15 min and plasma was immediately stored at −80°C. TNF-a and IL-6 were measured by enzyme-linked immuno-absorbent assay (ELISA), using a specific ELISA kit for equine Interleukin-6 (IL-6) (SEA079Eq, Cloud-Clone Corp., Katy, TX, USA) and for Tumor Necrosis Factor alpha (TNF-α) (SEA133Eq, Cloud-Clone Corp., Katy, TX, USA), according to specific manufacturer protocols.

### 2.4. Statistical Analysis

All statistical analyses were conducted using Prism 9 (GraphPad Software, LLC). All values are shown as the mean ± standard deviation (SD) of N observations (N = 8). Data were analysed by two-way ANOVA followed by Tukey’s multiple comparisons test. A *p*-value of less than 0.05 was considered significant.

## 3. Results

All ponies were included in the study. The body weight of the ponies was similar in both groups (group FL: 144 ± 22 kg and group A: 151 ± 18 kg). As reported in Table 1, there were no significant differences in body temperature, anaesthesia time, surgery time, and recovery time among the experimental groups.

**Figure 2:**
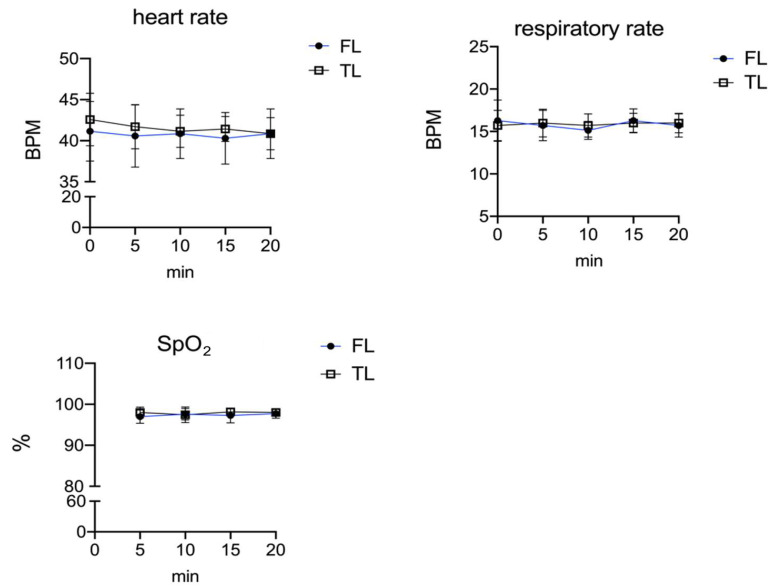
Timeline of heart rate (HR), respiratory rate (RR), and SpO_2_ during anaesthesia and surgical procedures. All values are shown as the mean ± standard deviation (SD) of N observations (N = 8) for each group.

We also found no significant difference in HR, RR, and SpO_2_ between the experimental groups as shown in Figure 2. In one animal, HR and RR were not recorded before sedation due to the nervous temperament of the pony. Three ponies, two in the FL group and one in the TL group, received additional sedation (0.3 mg/kg of IV xylazine) because sedation score was 1. No ponies showed an induction score of 0 or 1. Time from sedation to induction and quality of induction were similar in both groups. The anaesthesia time (time from induction to the end of the infusion), the time to perform the surgery (aseptic preparation, spermatic cord infiltration, and surgery), and the time from the end of the infusion to standing (recovery time) were similar in both groups (Table 1). The numbers of attempts to achieve standing position was two in one animal. The surgery was completed in all the animals, without anaesthetic or surgical complications. No animals required rescue analgesia postsurgery.

**Figure 3:**
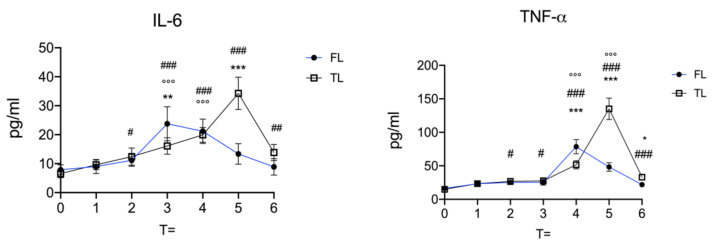
Timeline of IL-6 and TNF-α plasmatic levels measured by ELISA assay. FL, funicular group; TL, testicular group. All values are shown as the mean ± standard deviation (SD) of N observations (N = 8) for each group. # *p* < 0.05 versus T = 0 of TL group; ## *p* < 0.01 versus T = 0 of TL group; ### *p* < 0.001 versus T = 0 of TL group; °°° *p* < 0.001 versus T= 0 of TL group; * *p* < 0.05 FL versus TL; ** *p* < 0.01 FL versus TL; *** *p* < 0.001 FL versus TL.

As shown in Figure 1, at each time point blood samples were withdrawn for the determination of plasmatic levels of IL-6 and TNF-α. Figure 3 shows the timeline of the plasmatic concentration of IL-6 and TNF-α. In the preoperative basal state (T = 0), we found no statistical difference between the two groups in both IL-6 and TNF-α levels. During surgery (T = 2), only the group that received the intratesticular (TL) lidocaine showed an increase in both IL-6 and TNF-α compared to the respective baseline. During the recovery time one hour after the surgery (T = 3), we found a statistically significant difference between the two groups. Compared to the TL group, the FL group showed a higher level of IL-6. At this time point, both groups showed a significantly increased IL-6 level compared to the respective T = 0, and the TL group showed increased TNF-α levels from the respective baseline. The increased level of IL-6 from baseline was maintained for 4 h postsurgery (T = 4), but no statistical differences were observed between the two groups. The levels of TNF-α at T = 4 were significantly higher than baseline for both groups and when compared, the FL group showed higher levels of TNF-α. Eight hours postsurgery (T = 8), the TL group showed significantly increased levels of both IL-6 and TNF-α compared to baseline and to the FL group. At T = 8, the FL group showed an increased level from the basal line only for TNF-α. The last time point observed in this study was 24 h postsurgery T = 6. At T = 6, the FL group showed no statistical difference from baseline while the TL group showed a statistical increase from baseline levels of IL-6 and TNF-α and, when compared, the two groups at this time point showed a significant difference only in TNF-α levels, where the TL group showed the highest levels.

## 4. Discussion

The aim of this study was to evaluate the effect of intratesticular or intrafunicular locoregional lidocaine, to reduce perioperative nociception and cytokine release in ponies undergoing field castration under total intravenous anaesthesia. It is widely accepted that an optimal anaesthetic protocol is essential to achieve a good surgical outcome in terms of wound healing, pain, and local and systemic inflammation. In this regard, as already demonstrated, locoregional anaesthesia is a valid technique during surgical procedures to reduce the use of intravenous anaesthetic and reduce the perioperative nociception [9,23], as an important factor in surgical outcome and for patients’ well-being. To date, no study has been conducted on the direct comparison on the use of intratesticular or intrafunicular locoregional lidocaine in ponies undergoing surgical castration. In this study, fourteen healthy intact male ponies were scheduled for castration. Before castration, one group was injected with intratesticular lidocaine and the other received intrafunicular lidocaine. In this study, we focused on the plasmatic levels of IL-6 and TNF-α, as the two major cytokines able to drive inflammatory response, immune response, and pain sensitisation. IL-6 and TNF-α exert several physiological effects involved in immune and inflammatory response. In horses, elevated levels of IL-6 and TNF-α are related in several inflammatory diseases [24]. The time course of IL-6 and TNF-α secretion is also a key factor for surgical outcome. Inflammation is a physiological response after tissue injury and plays a key role in tissue repair, but an excessive and persistent inflammation, especially in the late postsurgical phases, is often related to a worsening of surgical outcome as well as increased postoperative pain perception [17]. Previous studies have shown that cytokines produced by non-neuronal cells can activate pain pathways in nociceptor neurons [25]. As already shown by several studies, both IL-6 and TNF-α are capable of inducing pain sensitisation, both locally and at central nervous system levels [26,27,28,29]. Thus, we aimed to compare the two locoregional anaesthesia techniques on the IL-6 and TNF-α production. In this study, we observed no statistical differences for IL-6 and TNF-α levels in the basal state T = 0. The increased production of IL-6 is a physiological response to tissue injury, but we observe a different pattern in IL-6 plasmatic level for intrafunicular or intratesticular lidocaine injection. For IL-6, the group treated with lidocaine injected intrafunicularly showed significantly increased levels one hour postsurgery T = 3. Subsequently, the levels decreased over the observation times and, starting from eight hours after surgery T = 5, were not statistically different from baseline. Additionally, the group injected with lidocaine intratesticularly showed a constant significant increase in IL-6 plasmatic levels after surgery, that reached a significant peak at eight hours after surgery, then the levels fell but remained significantly higher than baseline. Thus, these statistically significant differences in IL-6 levels observed at T = 6 as the “late phase” of this study, 24 h postsurgery, could indicate a different neuroendocrine response following the two different protocols of locoregional anaesthesia. Regarding the trend of TNF-α levels, no differences between groups were observed at T = 0 according to the IL-6 trend. Compared to the basal state, increased levels of TNF-α were observed four hours postsurgery in both groups. Four hours postsurgery, a peak in TNF-α level was observed for the intrafunicular group, at subsequent time points decreased levels were observed, and 24 h after the surgery no statistical differences were appreciable from baseline for the intrafunicularly treated group. While the group treated with testicular lidocaine showed a significant increment inTNF-α plasmatic level over time, a peak in TNF-α plasmatic levels was observed at 8 h after surgery, and at subsequent time points decreased levels were observed. Furthermore, the peak observed for the intratesticularly treated group was significantly higher than the peak observed for the intrafunicular group. According to IL-6 the trend, TNF-α levels at 24 h postsurgery were also statistically different from baseline for the intratesticular group.

These observations suggest that intrafunicular lidocaine or intratesticular lidocaine injection influence the trend of levels for IL-6 and TNF-α induced by surgical procedures. These observations could help in preventing postoperative complications and improve surgical outcomes and patient welfare during castration. The reduction of TNF-α levels minimises inflammatory and pronociceptive action of TNF-α [30], and the reduction of levels in IL-6 modulates the inflammatory pain induced by IL-6 [31]. As observed at 24 h postsurgery, statistical differences from baseline were observed only for the intratesticular group, where the cytokine levels were higher than baseline. It should also be noted that surgically damaged tissue can trigger cascading events that lead to the establishment of a neuroimmune loop, that can lead to the worsening or establishment of pathological conditions [26]. For example, cytokines released from surgically damaged tissue such as TNF-α and IL-6 induce local inflammation with activation of immune cells and neuronal sensitisation [32]. Therefore, beyond the neuroinflammation and the pain sensitisation, the neurons, once “activated”, can release several mediators themselves [33], that are able to stimulate the immune cells and establish a loop that leads to the worsening of clinical symptoms such as local and systemic pain [34]. For this reason, the importance of modulating the release of mediators such as TNF-α and IL-6 with appropriate pharmacological protocols could lead to an improvement of animal well-being [29], reducing pain and postoperative complications after field castration.

## 5. Limitation of the Study

The main limitation of the study is related to the sample size. Furthermore, a limiting factor could also be the limited end points investigated in this study. However, given the small amount of data present in this field to date, the results presented in this study provide important evidence on the impact of the different locoregional anaesthesia techniques on the immunological response in ponies undergoing field castration. Certainly, more studies will be needed to investigate more deeply the mechanisms underlying these responses in order to improve anaesthesia techniques and therefore animal welfare in field castration.

## 6. Conclusions

The results from this study suggest that the intrafunicular or intratesticular lidocaine locoregional anaesthesia influences the trend in IL-6 and TNF-α after castration surgery. Intrafunicular lidocaine locoregional anaesthesia is more efficient than testicular lidocaine locoregional anaesthesia in preventing surgically induced increased levels of IL-6 and TNF-α, in particular at 24 h postsurgery. These observations could also lead to a reduction of nociception and to minimising any inflammatory complications after surgery. Thus, intrafunicular lidocaine locoregional anaesthesia could be a useful technique in the anaesthesia protocol for field pony castration.

## Figures and Tables

**Table 1:** Anaesthesia and surgery time.

	FL	TL
TEMP °C	37.56 ± 0.395	37.6 ± 0.793
Anaesthesia time min.	44.29 ± 6.499	45.71 ± 6.395
Surgery time min.	25.86 ± 4.562	26.86 ± 4.980
Recovery time min.	38.49 ± 5.442	39.29 ± 3.638

## Data Availability

Not applicable.
